# CAREGIVING FOR PEOPLE WITH DEMENTIA DURING THE PANDEMIC: A SCOPING REVIEW

**DOI:** 10.1093/geroni/igac059.1947

**Published:** 2022-12-20

**Authors:** Phillip Cantu, Joanna Chyu, Neil Mehta, Markides Kyriakos

**Affiliations:** The University of Texas Medical Branch, Galveston, Texas, United States; UTMB, Galveston, Texas, United States; UTMB, Galveston, Texas, United States; UTMB, Galveston, Texas, United States

## Abstract

**Background:**

The COVID-19 pandemic has had a large impact on the roles and responsibilities of caregivers for older adults with dementia. An increasing number of studies have examined the unique challenges faced by caregivers during this time, including extended work hours, anxiety around contracting COVID-19, and adhering to public health guidelines. Informal caregivers may also face greater strain on their personal lives during lockdown. Objective: To conduct a scoping review to examine factors impacting well-being of caregivers of older adults with dementia during the first year of the COVID-19 pandemic. Method: We conducted a PubMed search using the terms “COVID-19,” “older adults,” and “caregiving” or “caregiver.” Sixty-seven papers were identified published between June 2020 and December 2021.

**Results:**

All papers identified were cross-sectional and conducted after the pandemic began and prior to the availability of COVID-19 vaccines. Articles highlighted increasing burdens such as financial and physical stress, as well as worsened psychological wellbeing through increased anxiety and depression among caregivers during the pandemic. Additionally, themes of protective factors on wellbeing in the form of social connection and telehealth interventions emerged. Discussion: Limitations to our review include lack of longitudinal information on caregiver experience to better identify broad impacts on caregiver well-being. Studies not only identified new, pandemic-related risk factors for caregiver burden, but also a heightened effect of pre-existing risk factors (e.g. income, living situation, gender) on burden. Caregiver psychiatric outcomes reflect the overall population’s increase in mental illness since the start of the pandemic.

